# Descriptive Usability Study of CirrODS: Clinical Decision and Workflow Support Tool for Management of Patients With Cirrhosis

**DOI:** 10.2196/13627

**Published:** 2019-07-03

**Authors:** Jennifer Hornung Garvin, Julie Ducom, Michael Matheny, Anne Miller, Dax Westerman, Carrie Reale, Jason Slagle, Natalie Kelly, Russ Beebe, Jejo Koola, Erik J Groessl, Emily S Patterson, Matthew Weinger, Amy M Perkins, Samuel B Ho

**Affiliations:** 1 Health Information Management and Systems The Ohio State University Columbus, OH United States; 2 Center for Health Information and Communication Richard L Roudebush Department of Veterans Affairs Medical Center Indianapolis, IN United States; 3 Department of Biomedical Informatics University of Utah Salt Lake City, UT United States; 4 Department of Biomedical Informatics The Ohio State University Columbus, OH United States; 5 Department of Veteran Affairs Salt Lake City Healthcare System Salt Lake City, UT United States; 6 Division of Epidemiology University of Utah Salt Lake City, UT United States; 7 Department of Veterans Affairs San Diego Healthcare System San Diego, CA United States; 8 Geriatric Research Education and Clinical Center Department of Veterans Affairs Tennessee Valley Healthcare System Nashville, TN United States; 9 Department of Medicine Vanderbilt University Medical Center Nashville, TN United States; 10 Department of Biomedical Informatics Vanderbilt University Medical Center Nashville, TN United States; 11 Department of Biostatistics Vanderbilt University Medical Center Nashville, TN United States; 12 Center for Research and Innovation in Systems Safety Vanderbilt University Medical Center Nashville, TN United States; 13 Department of Medicine University of California San Diego San Diego, CA United States; 14 Department of Family Medicine and Public Health University of California San Diego San Diego, CA United States; 15 Mohammed Bin Rashid University of Medicine and Health Sciences Dubai United Arab Emirates

**Keywords:** clinical decision support, human factors engineering, liver cirrhosis, interview

## Abstract

**Background:**

There are gaps in delivering evidence-based care for patients with chronic liver disease and cirrhosis.

**Objective:**

Our objective was to use interactive user-centered design methods to develop the Cirrhosis Order Set and Clinical Decision Support (CirrODS) tool in order to improve clinical decision-making and workflow.

**Methods:**

Two work groups were convened with clinicians, user experience designers, human factors and health services researchers, and information technologists to create user interface designs. CirrODS prototypes underwent several rounds of formative design. Physicians (n=20) at three hospitals were provided with clinical scenarios of patients with cirrhosis, and the admission orders made with and without the CirrODS tool were compared. The physicians rated their experience using CirrODS and provided comments, which we coded into categories and themes. We assessed the safety, usability, and quality of CirrODS using qualitative and quantitative methods.

**Results:**

We created an interactive CirrODS prototype that displays an alert when existing electronic data indicate a patient is at risk for cirrhosis. The tool consists of two primary frames, presenting relevant patient data and allowing recommended evidence-based tests and treatments to be ordered and categorized. Physicians viewed the tool positively and suggested that it would be most useful at the time of admission. When using the tool, the clinicians placed fewer orders than they placed when not using the tool, but more of the orders placed were considered to be high priority when the tool was used than when it was not used. The physicians’ ratings of CirrODS indicated above average usability.

**Conclusions:**

We developed a novel Web-based combined clinical decision-making and workflow support tool to alert and assist clinicians caring for patients with cirrhosis. Further studies are underway to assess the impact on quality of care for patients with cirrhosis in actual practice.

## Introduction

The burden of chronic liver disease (CLD) and cirrhosis on the US health care system is increasing [1]. CLD affects 30% of the US population [2], causing more than 36,000 deaths in 2014 [3]. CLD substantially reduces patients’ quality of life and leads to increased health care costs and indirect economic burdens [4]. While hepatitis C virus infection and alcohol-related liver disease still account for the majority of cirrhosis and liver transplants in the United States [5], nonalcoholic fatty liver disease is now the overall leading cause of CLD [6,7]. The prevalence of CLD is also increasing among veterans, mostly as a result of nonalcoholic fatty liver disease. CLD continues to place a heavy burden on the Department of Veterans Affairs (VA) system despite solid progress in reducing hepatitis C virus infections through antiviral treatment [8,9].

Despite research-based clinical care guidelines for cirrhosis [10], the treatment and quality of care for patients with cirrhosis are highly variable [11-13]. Factors affecting the adoption of guidelines for cirrhosis treatment include (1) failure to believe the available evidence as it applies to individual patients, (2) inadequate processes to inform clinicians about guidelines, (3) failure to exert the additional clinical effort to administer guidelines [14], and (4) reluctance to take on the additional cognitive load inherent in complex clinical care [15]. Strategies to improve the diagnosis and treatment of cirrhosis-related complications are difficult to implement, especially with the fast pace at which new clinical guidelines are made [15-17]. Our work focuses on clinical decision and workflow support tools within electronic health records (EHRs) that provide evidence-based guidance.

Early intervention is the best way to prevent the progression of cirrhosis to end-stage disease. Early-stage cirrhosis is frequently undiagnosed, however, until after there are clear manifestations of the disease [18,19]. Laboratory biomarkers and abdominal imaging can provide early indications of liver disease. Those tests are commonly undertaken in the inpatient setting for a variety of reasons but relevant abnormalities are frequently missed when a patient is under acute care for a non–liver-related issue.

Health information technology tools that provide clinical decision support (CDS) can aid clinicians caring for complex or unfamiliar patients [20-22]. Well-designed CDS can deliver information during the provision of care by aligning it with the clinical workflow [23-25]. The adoption of CDS tools may be hindered by sophisticated data requirements, poor user interface design, and poor integration into clinical work [21,26]. Previous studies have found that human factors engineering (HFE) can improve efficiency, reduce errors, increase technology adoption, and reduce early abandonment of CDS tools [27-30]. We used iterative user-centered design and formative evaluation to create Cirrhosis Order Set and Clinical Decision Support (CirrODS), a workflow and decision-support tool to aid in the identification and treatment of patients with cirrhosis.

## Methods

**Figure 1:**
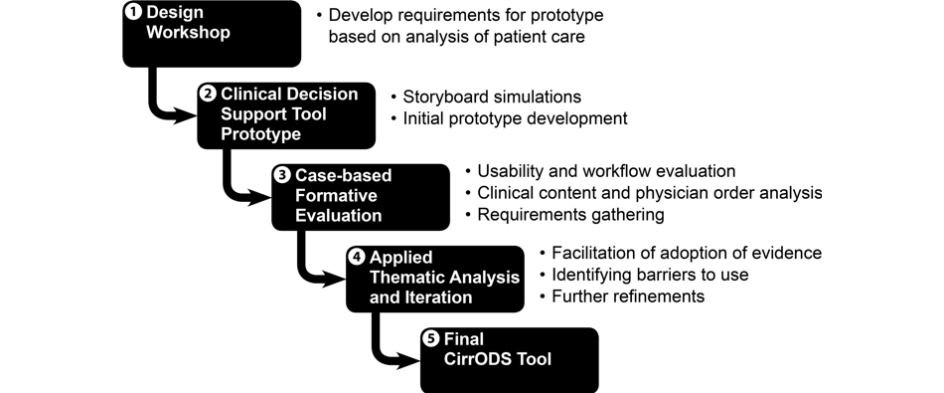
Design process. CirrODS: Cirrhosis Order Set and Clinical Decision Support.

The process to develop the CirrODS was composed of the five stages illustrated in Figure 1. We first identified the changes in clinical workflow that we wanted to achieve by using CirrODS. We then developed evaluation questions to assess how well CirrODS supported those changes during our formative evaluation procedures [31,32] (Multimedia Appendix 1, Table A). We then undertook iterative cycles of formative evaluation followed by design improvements [33].

### Ethics Approval and Consent to Participate

This work was reviewed and approved by the institutional review boards at Indianapolis VA Medical Center (1802327294), VA Salt Lake City Healthcare System (75714), VA San Diego Healthcare System (HI40012), and the VA Tennessee Valley Healthcare System (549271).

### Design Workshop

We convened a design workshop that included clinicians; user experience designers; information technologists; and health services, informatics, and human factors researchers from three VA clinical and research facilities. The workshop included eight clinical scenarios involving CLD [20,34]. The participants discussed the workshop goals and processes and then split into two groups, each of which worked through four of the scenarios (two inpatient and two outpatient). The clinician participants helped to clarify the scenarios so that the other participants understood the relevant clinical contexts and how the design principles might apply. Each group then selected two scenarios and drafted design storyboards using paper-based supplies [35,36]. The groups were then recombined and divided again to repeat the process. The scenarios that were not selected in the first round were reviewed in subsequent rounds. A common design concept emerged after three rounds of discussions and storyboard design.

### Cirrhosis Order Set and Clinical Decision Support Prototype Development

Following the storyboard simulations, our technology design team (consisting of a user experience designer, human factors experts, clinicians, informatics scientists, and a software engineer) created a CirrODS wireframe prototype. We used a controller layer and a persistence layer to support the user interface with test data from the Veterans Health Information Systems and Technology Architecture (VISTA) Integration Adapter, an application programming interface (API) approved by the VA for read/write access to VISTA [37]. We then employed a behavior driven development [38] framework to connect constituent pieces into use cases. Finally, we used unit-test driven development principles to create CirrODS [38-40].

### Case-Based Physician Order Analysis, Formative Evaluation, and Requirements Gathering

Subject matter experts (SMEs) created guideline-compliant clinical care orders that physicians could make in simulated patient-care scenarios. There were a total of 29 possible orders. Not all orders were appropriate for each scenario. The SMEs demarcated two levels of order appropriateness. First, guideline-meeting orders were orders that should be made for a specific scenario. Second, among the guideline-meeting orders, high-priority orders were orders that met grade IA evidence-based guidelines, defined as having health benefits based on data from multiple randomized controlled trials or meta-analyses [10]. We then undertook two rounds of semistructured interviews using a case-based, formative approach to develop and refine the CirrODS prototype.

In round 1, we interviewed two gastroenterology fellows and one internal medicine resident to enhance the initial clinical prototype and determine major changes in clinical content and data presentation. The physicians read four clinical scenarios involving ascites, gastrointestinal bleeding, encephalopathy, and compensated liver disease and interacted with screen shots of an initial CirrODS prototype to simulate the cognitive process of interacting with the tool to reach medical decisions. At the end of the session, the physicians completed the System Usability Scale (SUS) [41].

**Figure 2:**
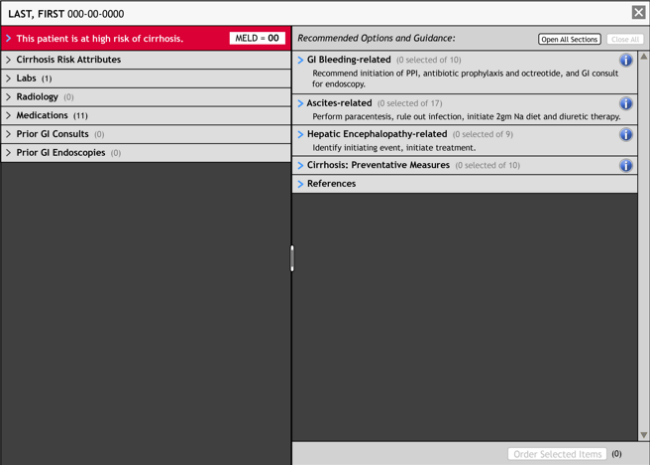
Initial clinical decision and workflow support tool prototype used in simulations. GI: gastrointestinal.

In round 2, 13 internal medicine residents and four interns from three VA medical centers read six clinical scenarios: two cases each of ascites, encephalopathy, and gastrointestinal bleeding. After reading the scenarios, the physicians made clinical care orders (consultations, medications, laboratory tests, radiology, other) specifically focusing on cirrhosis-related orders. For the first three scenarios (the control condition), the physicians reviewed the patients’ prior six months of medical records and wrote orders on paper without using CirrODS. For the next three scenarios, the physicians reviewed the patient records and formulated order plans using an interactive version of CirrODS (Figure 2). They then generated a paper copy of the orders using the same template as the control condition. We used a mixed-effects logistic regression model to determine if there were statistically significant differences between using the tool and not using it [41].

At the end of each round 2 session, the physicians completed the SUS [42] and the Electronic Health Record Usability Scale (EHRUS) [43]. The EHRUS is a 30-item usability scoring system designed to measure health care domain-specific concepts (ie, patient safety, quality of care, and continuity of care) in addition to the more traditional usability concepts (eg, efficiency, effectiveness, learnability) measured by the SUS. The EHRUS was designed to help interface developers identify potential areas of concern, particularly risks to quality of care, that would not be captured by the SUS. After completing the scenarios, the physicians were asked whether they recommended any changes or additions to the order choices.

### Applied Thematic Analysis and Iteration

Two individuals independently reviewed the transcripts of the semistructured interviews. We reviewed transcripts, identified snippets to inform redesign, iterated codes in 3 to 4 cycles to identify thematic domains, and then developed themes and recommendations using an applied thematic analysis [44,45]. The research team reviewed the recommendations (illustrated by snippets), refined them, and presented them to the design team.

### Refinement of the Cirrhosis Order Set and Clinical Decision Support Prototype

Our user experience designer constructed and iteratively refined [46] the prototype based on feedback from human factors experts, clinicians, and informatics scientists. In parallel, our software developer assessed the feasibility of the design. Access to EHRs through the API allowed real-time access to VISTA so that we could validate error logic and undertake quality control on the prerelease software using mock and test data from the EHR test environment. We used test-driven deployment as a harness to validate EHR API calls, comparing the results to equivalent requests made through the standard EHR user interface [37]. For quality assurance we undertook manual regression testing employing specific clinical use cases to validate the workflow. The research and design team iteratively refined the prototype over a period of about four months.

## Results

### Design Workshop

**Figure 3:**
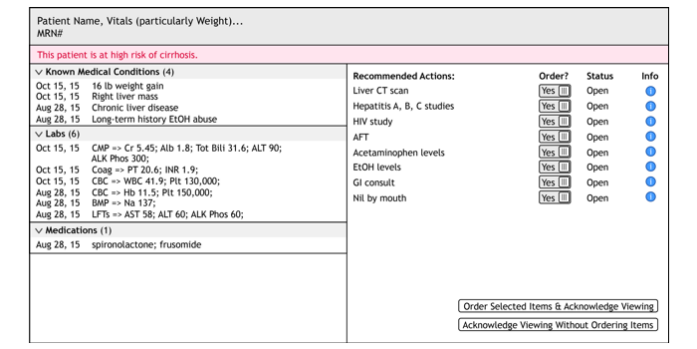
Example of concept design envisioned at the end of the design workshop.

Figure 3 shows the tool as envisioned at the end of the design workshop. The design reflects the relationships between the information used to assess cirrhosis (eg, causes, history, and physiological indicators) and the available interventions [47]. The assessment side (left side of Figure 3) includes patient demographics and information relevant to liver disease, such as radiology reports, medications, laboratory results, and consultations with specialists, with space for notes. The planning and action side (right side of Figure 3) includes interventions for specific cirrhosis-related problems such as gastrointestinal bleeding. The tool orders and organizes the information so that users can view disease progression over time.

### Case-Based Formative Evaluation, Order Evaluation, and Requirements Gathering

The average SUS score in round 1 was 75.8 [SD 3.0]. We used the feedback from the physicians in round 1 (Multimedia Appendix 2) to construct an interactive CirrODS prototype. We added information buttons, revised the content of the antibiotics orders, and added information about lower gastrointestinal bleeding. We improved access to ordering by permitting the user to hide/show evidence supporting the orders and to expand/hide all orders to permit easier viewing of sections. We also added a floating header and footer to provide reference to previous and next order groups. The revised screens were designed to be as close as possible to the Computerized Patient Record System (CPRS) used by the VA while providing decision support to the user. To do that, we updated the ordering to reflect the flexibility of CPRS ordering. We improved the fidelity in the patient search function by expanding the patient selection dialog to include additional details (eg, date of birth, social security number, gender, and last date of admittance). We updated the documentation that CirrODS creates in the CPRS to provide a clearer layout and to better reflect the orders made and manual orders not accessible by the system (eg, orders constrained by limitations in the interface, such as dietary orders). We also updated the order library to permit changes to what can be ordered based on evidence or clinical experience.

The physicians interviewed in round 2 were generally positive about the interactive prototype (Multimedia Appendix 2). When using the tool, physicians placed fewer orders overall and placed a higher percentage of orders in concordance with quality indicators compared with the orders placed when not using the tool (Table 1). Assessing the hepatic encephalopathy clinical scenario with the tool was associated with a higher percentage of guideline-concordant orders (52/104 [50.0%] vs 36/117 [30.8%], *P*=.004). The mean orders per participant were 13.66 (SD 12.85). The mean for orders meeting the guidelines was 46 in the control and 48 when using the CDS and 69 for control and 76 with the CDS for high-priority orders meeting the guidelines. Importantly, the number of participants was driven by the formative evaluation and not by a power analysis to test hypotheses. Because of this, future work could undertake a study with a larger sample size to determine if use of the tool results in statistically significant differences.

**Table 1:** Orders written with and without the clinical decision support tool in patient care simulations.

Cirrhosis-related condition^a^	Total number of orders per session		Orders meeting guidelines^b^			High-priority orders meeting guidelines^c^		
	Control, mean	Using CDS^d^, mean	Control^e^, n/N (%)	Using CDS^e^, n/N (%)	*P* value^f^	Control^e^, n/N (%)	Using CDS^e^, n/N (%)	*P* value^f^
Ascites (a)	17.11	15.13	79/126 (63)	65/112 (58)	.46	61/72 (85)	51/64 (80)	.51
Ascites (b)	16.5	12.78	50/120 (42)	56/135 (42)	.96	17/24 (71)	21/27 (78)	.57
Encephalopathy (a)	8.22	11.38	36/117 (31)	52/104 (50)	.004	26/63 (41)	39/56 (70)	.002
Encephalopathy (b)	14.25	13.88	57/112 (51)	56/112 (50)	.89	32/56 (57)	39/56 (70)	.20
GI^g^ bleed (a)	12.11	12.38	49/117 (42)	49/104 (47)	.43	29/36 (81)	25/32 (78)	.80
GI bleed (b)	13.75	11.56	53/112 (47)	52/126 (41)	.36	25/32 (78)	30/36 (83)	.59

^a^Two different patient scenarios (a and b) were used targeting each condition.

^b^Orders meeting guidelines: orders in which one or both subject matter experts considered the order relevant for that patient scenario at any grade level.

^c^High-priority orders: orders for which both subject matter experts considered the order relevant for that patient scenario in agreement with published cirrhosis quality measure guidelines [11,12].

^d^CDS: clinical decision support.

^e^Denominators are a product of the number of expected orders for the given scenarios and the number of participants who encountered the given scenarios.

^f^*P* value for fixed effect for CDS tool using a mixed-effects logistic regression model [41].

^g^GI: gastrointestinal.

Physician feedback on the appropriateness of the clinical content, patient information, workflow alignment, order set safety, awareness of cirrhosis indications, use of treatment evidence, and usefulness of the tool for decision-making is shown in Multimedia Appendix 1, Table B. In terms of the user interface, physicians indicated that the tool had good functionality and presented clinical content in a manner that improved efficiency (Multimedia Appendix 2). Multimedia Appendix 1, Table B, shows a summary of the suggestions made by the physicians in round 2 and the corresponding changes that were made based on our applied thematic analysis of the interviews. Multimedia Appendix 1, Tables C and D, show a full list of the modifications made to the tool after round 2 based on our applied thematic analysis of the interviews.

The average SUS score in round 2 was ** **78.2 [SD 11.9], indicating good usability [42]. The individual SUS item scores and the ratings of the EHRUS items are shown in Multimedia Appendix 1, Table E, with the items most relevant to the project’s design goals in bold [43]. Some of the items with the highest scores were related to patient safety, decision-making, and clinical practice standards (Multimedia Appendix 1, Table E, Elements 2, 4, and 12, respectively). Overall, the items that were most related to the design goals for the tool scored highly on the EHRUS, while the items with the lowest scores were not part of the design goals. For example, the EHRUS contains items about information sharing, which is not a priority for the tool. Other lower scoring items such as Elements 35 and 36 in Multimedia Appendix 1, Table E, will inform future refinement of the tool.

### Applied Thematic Analysis and Iteration

We identified three themes in the physician responses from the semistructured interviews. The first theme was a general appreciation for the design and features of the tool (Multimedia Appendix 2). The second theme was related to the appropriateness of the guidance provided by the tool for users with various levels of experience (Multimedia Appendix 2). CirrODS was perceived to best aid less experienced clinicians, serving as a double check for order completeness and facilitating the recognition of cirrhosis. Some interviewees indicated that it is important to find the right balance between providing meaningful guidance for inexperienced clinicians and not giving too much guidance for experienced clinicians. The third theme was related to the care setting and how it affects assessment and the placement of orders (Multimedia Appendix 2). There were different ideas about when the tool would be used in a clinical context, suggesting that it may be important to allow individual users to tailor the use of the tool to their workflow preferences.

### The Final Cirrhosis Order Set and Clinical Decision Support Tool

We used the recommendations gathered from the thematic analysis, order-set review, and usability assessments to make the final version of CirrODS, which has both active and passive CDS features. The CirrODS interface groups and displays preselected parameters to support decision-making. The tool provides active decision support by automatically calculating the Model for End-Stage Liver Disease score and providing alerts for high-risk patients [48]. The tool also has the capability to survey a given patient population for health care encounters (eg, emergency department visits or inpatient admissions). CirrODS is automatically updated as new information is entered into patients’ electronic health records. Other active features include predictive modeling and alerting to clinicians. The final tool is available as a Web-based interface. This clinical support framework is exportable for use in other VA medical centers and in additional EHR systems.

## Discussion

### Principal Findings

We iteratively evaluated and developed a CDS tool to improve the evidence-based management of cirrhotic patients during routine hospital practice by nonspecialists. The results gathered during initial evaluations were promising, and end users expressed interest and appreciation for CirrODS. Overall, the tool maintained good usability while facilitating the ordering of a higher percentage of high-priority measures compared with those ordered without the tool. We also demonstrated the usefulness of user-centered design to develop EHR-based CDS tools.

### Limitations

This work was undertaken in three VA medical centers with a limited number of clinical providers and gastroenterologists. The final CirrODS tool was designed for an inpatient setting. The technical framework is designed to be generalizable to other VA medical centers and other EHR systems with regard to the clinical content and the user interface layout. However, EHR data interchange API would have to be adapted to the source EHR. The EHR could make use of the Fast Healthcare Interoperability Resource (FHIR) standard by building an FHIR adaptor with the FHIR standard in the EHR. We plan to test CirrODS in an actual care environment in the near future.

This was a low-fidelity simulation study. The participants were not under the same cognitive and task loads that they would typically be under in a clinical environment. Furthermore, the participants knew that they were evaluating a cirrhosis CDS tool. Only one of the scenarios evaluated the case of cirrhosis as a secondary diagnosis. Thus, the participants were a priori focused on managing cirrhotic patients under dedicated (ie, no interruptions or distractions) lower workload and time pressure conditions. We believe that under actual clinical care conditions, the benefits of using CirrODS are likely to be greater, particularly when a cirrhotic patient has been admitted with a non–liver-related condition.

### Comparison With Prior Work

**Figure 4:**
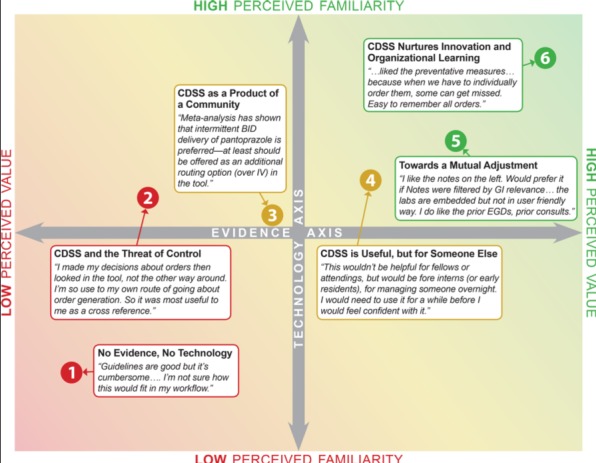
Examples from participant interviews of six positions representing perceived barriers and facilitators reported by Liberati et al [50]. CDSS: clinical decision support system.

Prior research suggests that attitudes toward CDS tools vary on the basis of clinicians’ attitudes or positions on specific scientific evidence and guidelines, interdisciplinary relationships, and organizational factors [49]. While we did not include an analysis of attitudes and positions about CDS in our formative evaluation, we found evidence in the interviews used for the applied thematic analysis that the physicians in our study articulated a range of such positions (Figure 4). Six positions were found to represent a gradient of perceptions representing barriers to CDS uptake and adoption. We used colors to ease visualization of differences in the positions. Green notes that end users of CDS perceives value and familiarity of the information. Positions in yellow note the CDS is viewed with some caution or concern. Red reflects positions that perceive CDS as a threat, challenge, or a problem. The first positions noted in red include clinician perceptions that the CDS may reduce their professional autonomy or may be used against them in the event of medical-legal controversies. In contrast, the positions in green reflect perceived value and good adjustment with regard to technical aspects and high usability [50].

This suggests that in addition to conducting a formative evaluation and usability assessment, it is important to assess the positions of the individual participants to inform the results of the evaluation. Future work on CDS tool development should assess the relevant positions of the individuals who participate in requirement gathering, formative evaluation, and preparation for implementation.

CirrODS shows the potential to support guideline-based care by facilitating the use of evidence-based order bundles for patients admitted for cirrhosis-related problems. The greatest value of CirrODS may be to help identify possible cirrhosis in patients under acute care for diagnoses unrelated to liver disease. In such situations, most acute care providers tend to defer nonacute management to future outpatient care, which may be delayed for weeks or months. Furthermore, if the nature and magnitude of liver disease are appreciated during hospitalization, physiological insults to the liver might be avoided or mitigated.

For example, if CirrODS identifies cirrhosis in a patient who is hospitalized for an infectious condition, the risk of further hepatocellular injury might be prevented by avoiding the use of hepatotoxic drugs to treat the infection. More generally, with the increasing emphasis on population health management, a tool that efficiently facilitates the delivery of evidence-based interventions to patients with early cirrhosis might substantially improve care quality and downstream outcomes.

### Conclusions

This work highlights lessons learned and user interface optimizations in alignment with user- centered design principles. We showed that although the sample size was modest in this evaluation, there was a significant increase in both appropriate ordering and high priority ordering for one of the test cases, a patient with cirrhosis and encephalopathy. Overall, our results suggest that the tool will enhance the performance of appropriate tasks and orders, serve as a double check for order completeness, and facilitate clinical decision-making by displaying relevant information. Further studies are needed to determine if CirrODS would result in measurable improvements in patient care and outcomes when used to treat patients in clinical settings.

## Appendix

Multimedia Appendix 1 Evaluation components, interview snippet results, and recommendations.

Multimedia Appendix 2 Snippets and themes.

## Abbreviations

API: application programming interface
CDS: clinical decision support
CirrODS: Cirrhosis Order Set and Clinical Decision Support
CLD: chronic liver disease
CPRS: Computerized Patient Record System
EHR: electronic health record
EHRUS: Electronic Health Record Usability Scale
FHIR: Fast Healthcare Interoperability Resource
HFE: human factors engineering
SME: subject matter expert
SUS: System Usability Scale
VA: Department of Veterans Affairs
VISTA: Veterans Health Information Systems and Technology Architecture
