# Supplementary material for: Descriptive Usability Study of CirrODS: Clinical Decision and Workflow Support Tool for Management of Patients With Cirrhosis
Source: JMIR Med Inform. 2019 Jul 3;7(3):e13627. doi: 10.2196/13627 (PMC6636234; doi:10.2196/13627)
Supplement: Multimedia Appendix 1 [file medinform_v7i3e13627_app1.docx]

**Multimedia Appendix 1**

**Appendix 1, Table A:** **Evaluation Questions**

| Evaluation Component | Evaluation Question |
| --- | --- |
| Guideline directed care | Was the clinical content relevant? |
| Support of macrocognitive work using HFE | Was the tool aligned with workflow? |
| Patient Safety | Were safe ordering practices presented? |
| Adoption and uptake of evidence using HIT | Did the tool increase awareness of the indications of liver cirrhosis and facilitate the use of treatment evidence as expressed in the ordering? |
| End user design effectiveness | Was the decision support tool useful? |
| Usability | Was the human computer interface well designed? |

**Appendix 1, Table B: Examples of snippets from Round 2 evaluation interviews**

| **Evaluation Area** | **Positive Snippets Related to Tool Design** | **Requirements, Recommendations, and Areas for Tool Redesign** |
| --- | --- | --- |
| Clinical content  (Assessing) | - *“I do like the prior EGDs, prior consults.”* - *“I like the references.”* | *“Orders I would add: procedural order set for paracentesis (PPE etc.).”*  *“Suggests total protein yet total protein is not an ordering option.”* |
| Alignment with workflow  (Informatics) | - *“Nice- like to close and open the carrots, could see what was ordered.”* - *“Yes, helpful; would be a timesaver in the long run.”* | *“I would never log in to this type of thing.”*  *“I did not like how I have to close the note to see the orders, too time consuming.”* |
| Safe ordering practice  (Ordering and Caring) | *“I didn't look at every single one (re: safety), the ones I saw were helpful.”*  *“Yes, tools are helpful to insure that we are more complete- and safety more thorough.”* | *“Wouldn't order IR for failure of endoscopic management.”*  *“Orders that might be unsafe: non-selective beta-blockers in some GI bleed situations.”* |
| Awareness of indications of liver cirrhosis and use of treatment evidence  (Diagnosing and Managing) | *“Yes- liked the preventative measures- HCC screening, vaccination, recommendation for antibiotics, prophylactic for SBP and treatment SBP- listed potential orders for paracentesis, because when we have to individually order them, some can get missed. Easy to remember all orders.”* | *“I didn’t know as much what I was going to do. This one led me along a path and may not have been the right thing this time. Might be dangerous…things to click as opposed to thinking as much.”* |
| Usefulness of the decision support tool  (Cross-issue) | *“I found it most helpful during the order process”*  *“So it was most useful to me as a cross reference.”* | *“Cirrhosis preventative care, I do not know what to make of it in this context.”*  *“Need more options- cirrhosis- need to be able to order NPO.”* |
| Human computer interface  (Informatics and Managing) | - *“I did find easy to navigate based on titles.”* - *“It’s a good tool, it pulls in related information without having to sort through CPRS.”* | *“It takes us longer to go thru the lab data when it is presented this way.”*  *“Just want to be able to add one thing and not redo the order. “* |

**Appendix 1, Table C:** **Recommended Tool changes and modifications**

| **Topic** | **Recommendation** | **Modification Made** |
| --- | --- | --- |
| Features favored by participants with additional requests | *“ I really like the MELD calculation and associated…(WAS NOT obvious to the user that the algorithm was available (MELD clickable)*  *“It’s a good tool, it pulls in related information without having to sort through CPRS. It’s a little unclear about labs. Overall I would use it if available.”* | Added indication to user interface to show that MELD score expands to provide additional details  Created additional view of labs to present in chronological order along with switch to permit changing between views (in progress at this time |
| Missing information/order sets | *“No dosing.”* | Dosing (as well as variable selections) endemic to ordering in a CPRS-like fashion added |
| Recommended changes in information/lab presentation | *“I liked the pertinent labs, disliked the labs organization. I’m not used to seeing them this way in this order, would prefer like panels in CPRS (most of us are used to worksheets). I was looking around for WBC, then INR they are out of order.”*  *“Change the order labs are displayed (should be displayed we are used to seeing in panels)”*  *“Would be more comfortable seeing all labs; hard to determine a select set of labs that would cover everything I need to know for every patient; would flip back to CPRS to see other labs in real world, worry what am I missing.”*  *“Labs: dates scroll of at the top, hard to have a comprehensive view over time.”* | Created additional view of labs to present in chronological order along with switch to permit changing between views.  Created additional view of labs to present in chronological order along with switch to permit changing between views.  Updating filter to show all versus specific results  Created additional view of labs to present in chronological order along with switch to permit changing between views |

**Appendix 1, Table D: Examples of requests and modifications made during evaluation**

| **Evaluation of Tool for Sociotechnical Dimensions** | **Interviews Requests** | **Description of Modifications Made** |
| --- | --- | --- |
| Software and Hardware | Assess alert function assessed with local IT infrastructure  Align tools with CPRS functionality | **Software**  -Date Range for EMR Search Added  -Open/Close order sections buttons consolidated  -Provided “expert” view to show/hide clinical recommendations  -If order made under one section, same order in all other sections updated to reflect selection  -Order dialogs completed to permit direct ordering within VistA, modeled after CPRS dialogs; integrated with VIA (VistA Integrated Adapter), Class I national API  -Updated feedback to user during data load from EMR (loading information screen) to provide indication of progress and data available  -Persisted Order Headings to float on top as user scrolls through orders to show where they are (in progress)  -Updated orders to reflect additions  **Hardware**  Migration to Windows 2012 Server to leverage IISNode hosting of NodeJS app to support multithreaded hosting |
| Clinical Content | Add Risk Attributes MELD / MELD Scores  **Snippets from Round 2 related to orders**  *“The routine GI Bleed labs I want to see are (Type & Cross screen, hemoglobin) not available in Tool.”*  *“I would ALSO like LAB PANELs specific for hepatology as an *ORDER SET (hard to find in CPRS and epic), there are a standard set that hep asks for and they are hard to find.”*  *“Orders I would add: procedural order set for paracentesis (PPE etc.)”*  *“Orders I would add are NPO diet; infectious workup; imaging: RU; GI Bleeding: CBC, renal, LFTs; discrim function, also for GI Bleed folate, multivitamin; FIB-4; thiamine. CIWA protocol; Alc Hep orders; IR biopsy.”*  **Snippets from Round 2 related to clinical information**   - *“When treating AKI need older labs to know the baseline kidney function.”* - *“I would like to access to actual (scanned) images of x-rays, EGDs.”* | MELD / MELDNa calculation updated to reflect update to model  Modifications were made in the order sets. |
| Human Computer Interaction | The participants in round 1 requested a presentation that could be opened or closed  **Snippets from Round 2 regarding human computer interaction**   - *“I liked the pertinent labs, disliked the labs organization. I’m not use to seeing them this way in this order, would prefer like panels in CPRS (most of us are use to worksheets). I was looking around for WBC, then INR they are out of order.”* - *“Change the order labs are displayed (should be displayed we are used to seeing in panels)”* | Accordions and Sub accordions  Created additional view of labs to present in chronological order along with switch to permit changing between views |

**Appendix 1, Table E: System Usability Scale (SUS) and Electronic Health Record Usability Scale (EHRUS) Items and Ratings**

| **Survey Item** | **Mean/Number of Responses** | **St. Dev.** |
| --- | --- | --- |
| *1.  I think that I could use the system without the support of a technical person.*  *(I think that I would need the support of a technical person to be able to use this system.)* | *4.9/17*  *(1.1)* | 0.2 |
| **2. The system supports appropriate practice standards for my role.** | 4.4/17 | 0.5 |
| *3. I could use the system without having to learn anything new.*  *(I needed to learn a lot of things before I could get going with this system.)* | *4.4/17*  *(1.6)* | 0.9 |
| **4. The system provides the information necessary to make correct decisions.** | 4.3/17 | 0.5 |
| **5. I would imagine that most people would learn to use this system very quickly.** | 4.2/17 | 0.6 |
| 6. It will be easy to teach co-workers to use this system. | 4.2/17 | 0.8 |
| 7. The system uses familiar terms, symbols, and units. | 4.2/17 | 0.8 |
| *8. I thought there was a lot of consistency in the system.*  *(I thought there was too much inconsistency in this system.)* | *4.2/17*  *(1.8)* | 0.8 |
| *9.  I found the system very intuitive.*  *(I found the system very cumbersome to use.)* | *4.2/17*  *(1.8)* | 0.8 |
| **10. I thought the system was easy to use.** | 4.1/17 | 0.9 |
| 11. I am able to do similar things the same way throughout this system. | 4.1/15 | 0.8 |
| **12. Using this system will improve patient safety.** | 4.0/17 | 0.7 |
| 13. I think that I would like to use this system frequently. | 3.9/17 | 0.9 |
| **14. This system will help me deliver higher quality patient care.** | 3.9/17 | 0.7 |
| 15. The system's various features and functions work well together. | 3.9/17 | 0.9 |
| **16. I will be able to use this system effectively when working under pressure.** | 3.9/17 | 0.7 |
| 17. The system does a good job of protecting patient privacy. | 3.9/14 | 0.8 |
| **18. The system provides only necessary prompts.** | 3.9/15 | 1.1 |
| *19.  I found the system to be simple.*  *(I found the system unnecessarily complex.)* | *3.9/17*  *(2.1)* | 0.8 |
| 20. I felt very confident using the system. | 3.8/17 | 0.8 |
| 21. It is easy to find the information I need in this system. | 3.8/17 | 0.6 |
| 22. I will remember how to perform infrequent tasks with this system. | 3.8/17 | 1.2 |
| 23. I can quickly enter data into this system. | 3.8/14 | 1.2 |
| 24. The system is well designed for people who do my job. | 3.8/17 | 0.8 |
| 25. I am confident that I can use this system to do my job. | 3.7/17 | 0.8 |
| 26. The system makes it easy to identify the next step in a task. | 3.7/16 | 0.9 |
| 27. The system will prevent mistakes. | 3.7/17 | 0.8 |
| 28. The system contains only necessary features. | 3.7/17 | 0.7 |
| 29. I found the various functions in this system were well integrated. | 3.6/17 | 0.9 |
| 30. I can easily correct mistakes with this system. | 3.6/16 | 1.1 |
| 31. The system makes it easy to confirm I am working in the correct patient's record. | 3.6/16 | 1 |
| 32. Using this system will decrease my workload. | 3.4/17 | 1 |
| **33. The system provides valuable alerts.** | 3.4/13 | 1.3 |
| 34. The system rarely requires me to enter the same information more than once. | 3.3/15 | 0.8 |
| 35. The system allows me to complete tasks in my preferred order. | 3.2/17 | 1 |
| 36. The system makes it easy to detect how a patient's health has changed over time. | 3.2/17 | 1.2 |
| * The system gives me a comprehensive view of a patient's health. | 3.0/17 | 1.1 |
| * The system supports communication during patient hand-overs. | 2.9/8 | 1.5 |
| * The system makes it easy to share information with other health care professionals. | 2.8/10 | 0.9 |
| * The system makes it easy to share information with patients. | 2.8/9 | 1.3 |

Underlined = individual SUS items (for comparison)

*Italics* = negatively framed items converted to positively framed items (original item wording in parentheses)

**Bold** = Items aligned with specific project design goals

* = These items measure attributes that were irrelevant to or not a priority for the CDS design.

Note: For comparison purposes, the negatively framed SUS items (i.e., a low score reflects good usability) have the wording and mean score inverted [49] (original SUS mean score is listed in parentheses) to facilitate comparison with the positively framed items (i.e., a higher score reflects good usability) from the SUS and EHRUS. The scale for all items is from 1 (Strongly Disagree) to 5 (Strongly Agree).
