# Supplementary material for: Descriptive Usability Study of CirrODS: Clinical Decision and Workflow Support Tool for Management of Patients With Cirrhosis
Source: JMIR Med Inform. 2019 Jul 3;7(3):e13627. doi: 10.2196/13627 (PMC6636234; doi:10.2196/13627)
Supplement: Multimedia Appendix 2 [file medinform_v7i3e13627_app2.docx]

**Multimedia Appendix 2**

**Snippets from Participants, and Themes from Applied Thematic Analysis**

Supporting Snippets For Section 3.2 *Case-based formative evaluation, order evaluation, and requirements gathering*

We made the following revisions to CirrODS after the Round 1 evaluation interviews, and the snippets below reflect the need for modifications:

We added info buttons, *“the roll-over INFO buttons are insufficient and inconvenient for the user to see the ordering rationale”*.

We also revised the content of the antibiotics orders, and added information about lower GI bleeding, “*this particular GI bleeding pathway seems totally focused on variceal bleeding, doesn’t include anything about lower GI bleeding”* and, *“Prophylactic antibiotics for SPB prevention, should add Rocephin – it’s the most common given. Alternate is Bactrim, which is helpful if they have an allergy for the fluoroquinolones.”*

In Round 2, the following snippets reflect that the refined prototype was generally well received by clinicians (See section 3.2).

*“I do like the prior EGDs, prior consults.”*

*“I like the references.”*

*“Nice- like to close and open the carrots, could see what was ordered.”*

*“Yes, helpful; would be a timesaver in the long run.”*

*“I didn't look at every single one (re: safety), the ones I saw were helpful.”*

*“Yes, tools are helpful to insure that we are more complete- and safety more thorough.”*

Supporting Snippets For Applied Thematic Analysis and Iteration

We found several themes based on the supporting snippets. The themes relate to workflow, clinician experience, and timing of the optimal timing of the use of CirrODS. The themes inform future iteration of the workflow tool, the need to tailor use of the tool, training materials, and as well timing of use during implementation.

Theme: CirrODS is useful to clinicians in terms of design and specific features.

*“I would use this voluntarily or if offered in another way.”*

*“Nice- like to close and open the carrots, could see what was ordered.”*

*“It’s a good tool, it pulls in related information without having to sort through CPRS.”*

Theme: CirrODS supports clinical workflow from novice to expert.

“Early stage, when you know you have a cirrhotic, maybe after hours, but too late after hepatology consult.”

“Early trainees most likely to use.”

“Bypasses some of general problems we encounter in CPRS... validates order sets, so I would teach my intern to use and trust.”

Theme: Clinicians describe varying points in the workflow for CirrODS use.

*“When we are admitting the patient is most usable.”*

*“When I do H&P, that’s when I’m doing all these orders.”*

*“Would access after initial consult.”*

*“I’d want to use it upon order entry in CPRS, wouldn’t want to see it right away upfront. I found it most helpful during the order process.”*
